# IN-HOME ASSESSMENT OF SUICIDALITY IN RURAL-DWELLING OLDER ADULTS

**DOI:** 10.1093/geroni/igae098.1837

**Published:** 2024-12-31

**Authors:** Caitlyn Nix, Branden Schaff, Mary Dozier

**Affiliations:** Mississippi State University, Mississippi State, Mississippi, United States; MIssissippi State University, Starkville, Mississippi, United States; Mississippi State University, Mississippi State, Mississippi, United States

## Abstract

Older adults in isolation have an increased risk for suicidality, and this can be exacerbated in a rural context. Additionally, assessment of suicidality during in-home care for older adults is different from typical clinical settings. This presentation will focus on first-hand clinical experiences from an ongoing clinical trial for older adults with hoarding disorder (n = 44, mean age = 69) in rural Mississippi. All participants completed a semi-structured interview of suicidality risk at baseline and at follow-up points across the intervention. We will discuss various factors that are unique to a rural-in home assessment setting, including the importance of re-emphasizing information critical to informed consent (e.g., thresholds for breaking confidentiality), sensitivity to the vulnerability of participants being asked such questions within their home, and confidentiality concerns (e.g., cohabitants in the home, procedures for contacting supervisor for consultation). As the individuals in the current study were volunteering due to distress from clutter or items in the home and not active suicidal ideation, conclusions from these clinical experiences will be compared with findings from risk-assessment studies in older adults.

